# Gender score development in the Berlin Aging Study II: a retrospective approach

**DOI:** 10.1186/s13293-020-00351-2

**Published:** 2021-01-18

**Authors:** Ahmad Tauseef Nauman, Hassan Behlouli, Nicholas Alexander, Friederike Kendel, Johanna Drewelies, Konstantinos Mantantzis, Nora Berger, Gert G. Wagner, Denis Gerstorf, Ilja Demuth, Louise Pilote, Vera Regitz-Zagrosek

**Affiliations:** 1 Berlin Institute for Gender in Medicine, Charité – Universitätsmedizin Berlin, Berlin, Germany; 2 CCR (Centre for Cardiovascular Research) Berlin, Berlin, Germany; 3 DZHK (German Centre for Cardiovascular Research), partner site Berlin, Berlin, Germany; 4 Centre for Outcomes Research & Evaluation, Research Institute of the McGill University Health Centre, Department of Medicine, McGill University, Montreal, Canada; 5 Gender in Medicine, Charité – Universitätsmedizin Berlin, Berlin, Germany; 6 Department of Psychology, Humboldt-Universität zu Berlin, Berlin, Germany; 7 Medical Clinic for Endocrinology, Biology of Aging group, Charité – Universitätsmedizin Berlin, Berlin, Germany; 8 SocioEconomic Panel at the German Institute for Economic Research (DIW), Berlin, Germany; 9 Charité - Universitätsmedizin Berlin, BCRT - Berlin Institute of Health Center for Regenerative Therapies, Berlin, Germany; 10 Department of Medicine, McGill University, Montreal, Canada; 11 Department of Cardiology, University Hospital Zurich, Zurich, Switzerland

**Keywords:** Gender, Sex, Aging, Berlin Aging Study II, BASE-II, Gender score

## Abstract

In addition to biological sex, gender, defined as the sociocultural dimension of being a woman or a man, plays a central role in health. However, there are so far few approaches to quantify gender in a retrospective manner in existing study datasets. We therefore aimed to develop a methodology that can be retrospectively applied to assess gender in existing cohorts. We used baseline data from the Berlin Aging Study II (BASE-II), obtained in 2009–2014 from 1869 participants aged 60 years and older. We identified 13 gender-related variables and used them to construct a gender score by using primary component and logistic regression analyses. Of these, nine variables contributed to a gender score: chronic stress, marital status, risk-taking behaviour, personality attributes: agreeableness, neuroticism, extraversion, loneliness, conscientiousness, and level of education. Females and males differed significantly in the distribution of the gender score, but a significant overlap was also found. Thus, we were able to develop a gender score in a retrospective manner from already collected data that characterized participants in addition to biological sex. This approach will allow researchers to introduce the notion of gender retrospectively into a large number of studies.

**Supplementary Information:**

The online version contains supplementary material available at 10.1186/s13293-020-00351-2.

## Introduction

Women and men differ in disease manifestations and outcomes though the underlying mechanisms are still poorly understood [1, 2]. Yet, it is well known that sex and gender play a central role in health [3–7]. Sex is the biological condition of being male or female and includes differences in genetic and hormonal levels. Its role in major diseases is well documented. In contrast, gender incorporates the psychological, behavioural, social, and cultural aspects, i.e. the sociocultural dimension of being a woman or a man in a given society [8–10]. Gender is composed of four dimensions, reflecting different aspects, e.g. gender roles, gender relations, institutionalized gender, and gender identity [11, 12]. Gender roles represent the behavioural norms applied to men and women in society, which influence individuals’ everyday actions, expectations, and experiences. Gender identity describes how we see ourselves as women or men (or as a third gender) and affects our feelings and behaviours. Gender relations refer to how we interact with or are treated by people in the world around us, based on our ascribed gender. Finally, institutionalized gender reflects the distribution of power between women and men in the political, educational, and social institutions in society.

The definition of gender has changed over time, but most researchers in the present times follow the definitions above that also underlie the definitions at the homepage of Canadian Institutes of Health Research and the National Institutes of Health, US and Horizon 2020, that promote the largest research programs in gender medicine [9]. Since biological sex and gender overlap, but are not identical, gender is likely to influence health differently from biological sex [3, 4].

Gender encompasses awareness of disease, risk taking and help-seeking behaviour, interaction of patients with doctors, the health care system [13, 14], and access to care. Gender, for example, is associated with a later arrival of women with myocardial infarction to the emergency departments and delayed access to treatments, as well as different treatment results by female and male doctors in their female and male patients [14].

To separate the effects of both sex and gender, variables that represent both in multivariate models are needed. Biological sex is usually assessed by a single binary variable on a one-dimensional scale (female, male) assuming that the sex chromosomes are unique identifiers of female and male individuals, even though this concept has been revised—resulting in the German legal system allowing for coding the sex of new-born babies to be undetermined, which might be a better reflection of biology. However, so far, the binary coding of sex has proved itself as a useful construct in clinical studies to describe differences between female and male individuals.

Unfortunately, there is no generally accepted method to assess the sociocultural dimension that is gender. Thus, researchers need to identify a number of gender-related variables and include them in multiple regression models. Depending on the number of events predicted, it may be impossible to include all aspects of gender in model. Compared to this approach, the use of a composite score measuring gender could offer advantages. Such a score can be used for adjustment in multiple regression models where a distinction from sex is the primary goal, for matching, and subgroup stratification in order to better control confounding variables [15, 16]. As compared with the use of a set of gender-related variables, a single score provides greater statistical power by reducing the number of covariates included in multiple regression models, offer the possibility to test interaction terms, and reduce multiple comparisons [15, 16].

In the present paper, we therefore searched to develop a method to operationalize gender in the context of clinical studies by developing a gender score, following previously published concepts [3, 17, 18].

In 2015, the Canadian members of our group constructed a gender score that was obtained by a questionnaire in a prospective manner and showed that this gender score was associated with health outcomes, in particular survival after acute coronary syndrome. This gender score was based on a variety of carefully selected psychosocial and sociocultural variables [3, 4]. In a prospective study in patients with acute coronary syndrome, the gender score was more strongly associated with several important cardiovascular risk factors, such as diabetes, hypertension, and family history for cardiovascular diseases and 1-year recurrent events than biological sex [3, 4]. It was concluded that a gender score can be used to evaluate the effect of gender beyond the effects of sex on disease risk, presentation, processes of care, and relevant outcomes. Including such a gender dimension into clinical studies may thus add new explanatory value to understand differences between women and men in disease manifestations and outcomes.

However, the majority of existing studies and databases did not include a gender questionnaire and did not measure gender in a prospective manner. Consequently, it is necessary to develop a methodology to assess gender in a retrospective approach in existing datasets. Since most study databases include a large number of psychosocial and/or socioeconomic variables that are potentially gender related, it is possible to construct a score to measure gender. Two recent studies have done this [17, 18]. In one study, a large population-based database was used, the Canadian Labour Force survey, to extract the most suitable variables related to the gender dimensions described above and to derive a gender score from these variables [18]. Most variables available in this dataset were related to work and living conditions, with a focus on gender roles and institutionalized gender [17, 18].

However, the fact that two of the four dimensions of gender, gender relations and gender identity, were only poorly covered in these scores represents a limitation. Furthermore, a longitudinal study to test the impact of gender on health outcomes was not possible due to their cross-sectional design, representing another limitation. The fact that both scores are only reflecting the Canadian workforce and society is another limitation to their use in Europe.

To overcome these limitations in the previous work and to develop a gender score for a European population, one that was not solely focused on work conditions and that offered the opportunity to assess the impact of gender on clinical outcomes, we decided to use, in a paradigmatic approach, the Berlin Aging Study II (BASE-II) to construct a gender score in a retrospective manner. We selected potentially gender-related variables from the initial investigation 6–10 years ago that were closely representing the gender-related variables used in the GENESIS-PRAXY study and in the studies by Smith and Lacasse and that covered the above mentioned four different dimensions of gender (Table 1) [17, 18]. Gender identity was operationally approached using chronic stress and perceived stress, as done also by Lacasse [17], by the Big Five personality traits [19], and risk-taking behaviour [20]. Gender roles were operationally defined using employment status. Gender relations were represented by loneliness and institutionalized gender was represented by education and family status. Due to the fact that data assessment was done from 2009 to 2014, i.e. 6–10 years ago, and the study participants have been medically reinvestigated from 2018 to 2020, we are able to test the association between gender measured 6–10 years ago with more recent clinical status and outcomes in a longitudinal approach.

**Table 1:** Selected gender-related variables from the BASE-II cohort

Mean (SD)	Female (***n*** = 543)	Male (***n*** = 502)	***p*** value	Gender dimension
Age	68.4 (3.4)	68.8 (3.5)	0.06	n/a
Diabetes mellitus type II, *n* (%)	54 (9.9)	79 (15.7)	0.007	n/a
Hypertension, *n* (%)	250 (46.0)	242 (48.2)	0.05	n/a
Current smoking, *n* (%)	38 (7.0)	58 (11.6)	< 0.001	n/a
Myocardial infarction, *n* (%)	6 (1.1)	14 (2.8)	0.05	n/a
Risk-taking behaviour	3.05 (1.42)	2.43 (1.65)	< 0.001	Gender identity
PSS	1.52 (0.35)	1.46 (0.34)	< 0.001	Gender identity
TICS	1.36 (0.67)	1.11 (0.63)	< 0.001	Gender identity
BFI: Extraversion	5.07 (1.2)	4.78 (1.17)	< 0.001	Gender identity
BFI: Conscientiousness	5.6 (0.9)	5.50 (1)	0.01	Gender identity
BFI: Agreeableness	5.3 (1.03)	5.13 (0.96)	< 0.001	Gender identity
BFI: Neuroticism	3.76 (1.33)	3.36 (1.25)	< 0.001	Gender identity
The good things in my life	n/a	n/a		Gender identity
BFI: Openness to experience	5.09 (1.18)	4.97 (1.18)	0.05	Gender identity
UCLA-Loneliness	3.05 (0.34)	3.01 (0.33)	0.03	Gender relations
Employment status 2014	n/a	n/a		Gender roles
Family status 2009–2014	n/a	n/a		Institutionalized gender
Education	13.7 (2.76)	14.37 (1.4)	< 0.001	Institutionalized gender

Each variable was taken according to the definition provided in the Women health research network [11]. *MI* myocardial infarction, *PSS* perceived stress scale, *TICS* Trier Inventory for Chronic Stress (TICS), *BFI* big five Inventory, *UCLA* University of California, Los Angeles (*n* = 1045)

We hypothesized that it is possible to construct a gender score with this approach in a retrospective manner that distinguishes women and men based only on sociocultural factors and that integrates a number of gender-related variables. We hypothesized that the gender score overlaps with sex but differs from biological sex and gives additional information. We assumed that a number of males would have feminine gender characteristics and vice versa and that a significant overlap would exist, representing individuals with characteristics of both genders. We also assumed that the distribution of this gender score between females and males would be similar but not identical to the distribution of a gender score developed by our partners in Canada in the GENESIS-PRAXY study, and we assumed that sex and gender would be differently associated with clinical parameters. Associations with 6–10 years follow-up data are planned for the near future.

## Methods

### Data source

We used baseline data from the Berlin Aging Study II (BASE-II). Clinical, psychosocial, and socioeconomic variables were obtained in 2009–2014 from 1869 participants aged 60 years and older [21]. Selection of gender-related variables was done in 2018 (see below). Sex was determined by both self-reports and records in the official registry offices. For all but one of the participants used in the current data set, self-reports and official registry information converged. For one person, no official registry information could be obtained. Self-reports and registry information were further corroborated based on genetic data available for 98.2% of our sample (data not shown).

### Identification of gender-related variables

For identification of gender-related variables, we, as previous investigators have done the following: (a) referred to the systematics and gender dimensions proposed by Johnson and (b) included variables that have been proposed by other researchers in the field [3, 12, 16, 17]. First, we selected potentially gender-related variables from the study database related to the four dimensions of gender (Table 1 and supplement S1) that reflect different aspects of the gender construct in which women and men traditionally differ and that have been used by other investigators in the field [11, 12]. To the extent possible, the four gender dimensions were measured using validated self-report questionnaires [22–25]. Chronic stress was assessed using the Trier Chronic Stress Inventory; perceived stress with the Perceived Stress Scale [22]; the personality dimensions openness to experience, conscientiousness, extraversion, agreeableness, and neuroticism with a short version of the Big-Five Inventory [19]; loneliness using the UCLA Loneliness Scale [23]; and single-item questions for risk-taking behaviour [20], employment status, family status, and education. When possible, we calculated scores according to questionnaire standard. For the Big-Five Inventory, Trier Chronic Stress Inventory, and UCLA-Loneliness, mean scores across items were used, as used in previous publications [22, 24, 26]. The item “The good things in my life are determined by other people” is a standard item assessing external control beliefs in powerful others [27–29].

All participants gave written informed consent. The Ethics Committee of the Charité – Universitätsmedizin Berlin approved the study (approval number EA2/029/09) [30].

### Overall strategy and statistical approach

**Fig. 1:**
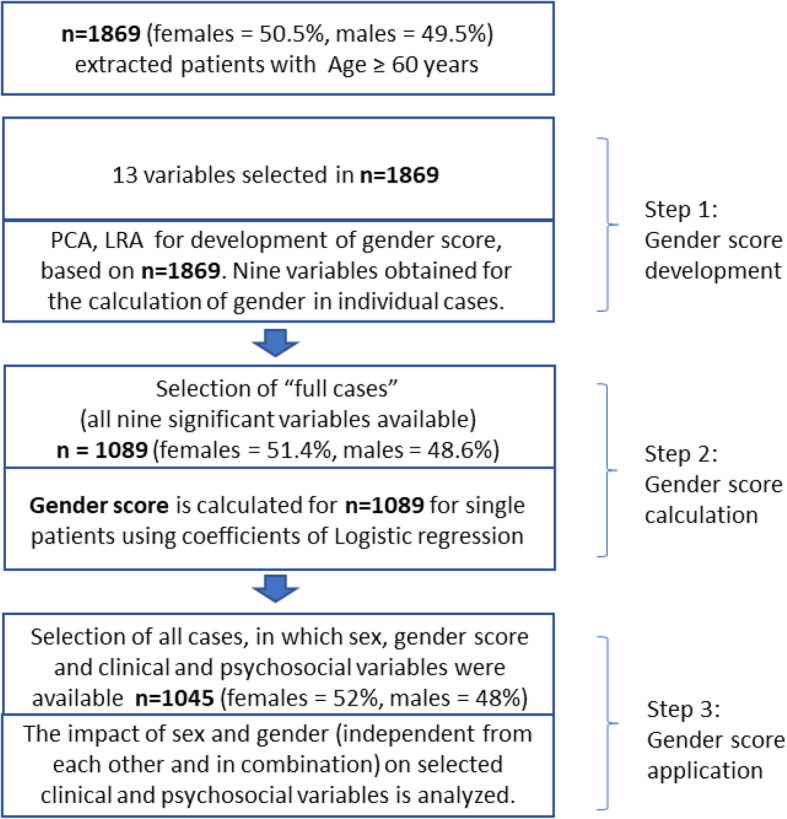
Stratification of data analysis. Stratification of data analysis was divided into three major steps. In step 1, the algorithm for the gender score was developed; in step 2, gender score was calculated for individual cases; and in step 3, the implication of the gender score was estimated

Our approach was divided into three steps (Fig. 1): (1) gender score development, (2) gender score calculation in individual cases and distribution in females and males, and (3) correlation with clinical variables.

#### Gender score development

In step 1, correlated variables were eliminated using a bivariate analysis, and for each correlated pair of variables with a correlation coefficient equal or greater than 0.80, one of the two variables was randomly removed (Table S1). Next, to reduce the dimensionality of the dataset, increase interpretability, and minimize information loss which is done by creating new uncorrelated variables and maximize variance, principal component analysis (PCA) was conducted with a varimax rotation method to further confirm it, where component represents the linear combinations of the entered variables and each component represents a group of related variables [31]. An item was said to load on a given component if the factor loading was 0.40 or greater for that component and was less for other components. We identified 5 major components (Table 2) and retained all 13 variables as the factor loading for the given two components of the same variable did not exceed 0.40. Variables having low communalities (< 0.40) do not contribute much to measuring the underlying factors [32].

**Table 2:** Gender-related variables and corresponding factor loading in PCA

	Component				
	1	2	3	4	5
Employment status 2014	− .041	− .082	− .097	− .042	**.713**
Risk-taking behaviour composite	− .004	.122	.384	**.592**	− .137
BFI-Neuroticism	**.787**	− .093	.047	− .068	− .002
BFI-Agreeableness	− .169	.297	− **.486**	.067	.066
BFI-Extraversion	− .057	**.767**	− .092	− .041	.062
BFI-Conscientiousness	− .227	**.455**	− .386	− .057	− .129
BFI-Openness	− .012	**.812**	.088	.060	− .021
The good things in my life	.381	− .145	.114	− .057	**.403**
Perceived stress	**.815**	− .092	− .024	.027	− .076
Chronic strain	**.819**	.016	.013	.106	− .056
UCLA-Loneliness: composite	− .176	.323	.121	.079	**.567**
Family status 2009–2014	.045	− .089	− .256	**.823**	.080
Education	− .100	.092	**.730**	− .011	.060

In this factor analysis, an item was said to load on a given component if the factor loading was 0.40 or greater for that component and was less than 0.4 for other components. Based on this analysis, all items were included in logistic regression (*n* = 1869)

To determine how the remaining sociocultural variables differentiated between females and males, logistic regression models for the association with sex were calculated one by one and all non-significant variables were removed in descending order of their significance. Coefficients of this logistic regression analyses were used to construct a propensity score [33] which was called gender score, in the same approach as used by previous investigators [3, 17, 18]. For step 1, the development of the algorithm for the gender score, the selected 13 variables were used in the dataset of 1869 cases.

#### Gender score calculation in individual cases and distribution in female and male

**Fig. 2:**
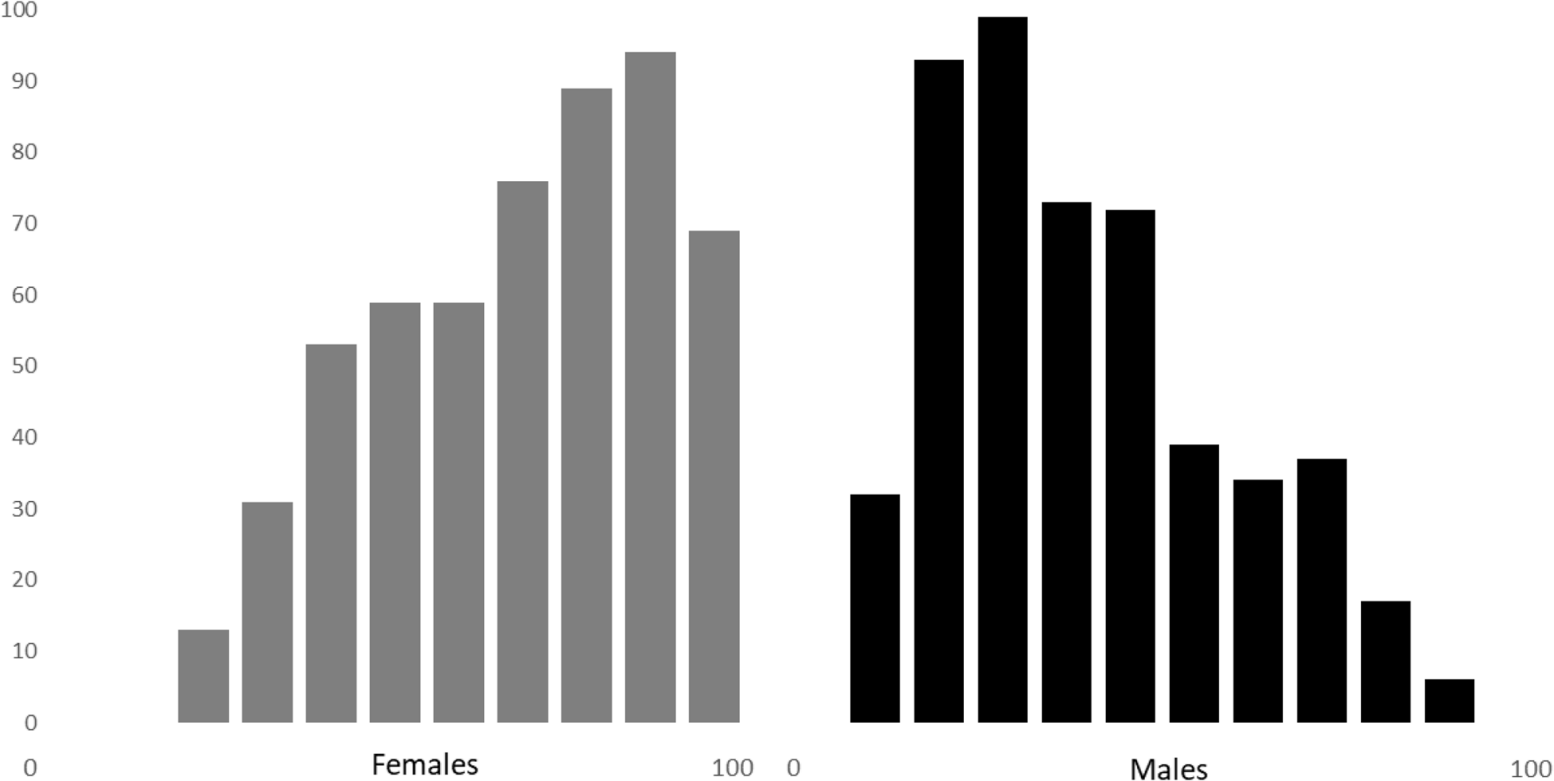
Gender score distribution in females and males. Distribution of calculated gender score in females (*n* = 543) and males (*n* = 502). Zero is identical with completely masculine characteristics whereas 100 represents completely feminine characteristics

In step 2, the coefficient estimates of the variables from logistic regression analyses were used for gender score calculation in individual cases, i.e. a score from 0 to 100 was obtained for all individual cases. For this step, the presence of all variables was required in each study subject. This reduced the number of cases available for calculation of individual gender score to 1089 (Fig. 1). Next, we analysed the distribution of this gender score with regards to biological sex (Fig. 2).

#### Association of gender score with clinical variables

In the third step, the association of gender score with independent clinical and psychosocial variables that were generally attributed to women and men in our societies was tested using linear regression analyses. This step required the presence of gender score and the clinical/psychosocial investigation in all cases. This reduced the number of cases to 1045.

#### Statistical tests

SPSS version 25 was used to perform statistical analysis. Logistic models were used to model the probability of associated variables. To look for an index of sensitivity and specificity, the curve statistic was performed for gender score (0–100) as the test variable and sex (0 = males, 1 = females). Significance level was determined for *p* < 0.05 for the group differences and logistic regression models.

## Results

### Development of the gender score

Mean age, sex, and basic clinical coordinates of our cohort are presented in Table 1. To calculate the gender score in our cohort, we considered a total of 13 variables (Table 1). In the first step of gender score development, correlation and principal component analysis correlation matrix for the variables was low, and therefore, all 13 variables were retained for the next steps (Table 2, PCA, factor loadings).

In the second step, the 13 identified sociocultural variables were used to perform a logistic regression analysis with sex as a dependent variable. Non-significant variables were removed one by one in a descending order of their *p* value (0.05). In total, five models were explored. The final model (Table 3) identified nine statistically significant sociocultural variables that were assumed to characterize women and men in our model. They were chronic stress, marital status, risk taking behaviour, Big-Five personality traits scale (agreeableness, neuroticism, extraversion), loneliness (UCLA-Loneliness mean), Big-Five personality traits scale (Conscientiousness), and level of education.

**Table 3:** Gender-related variables for the calculation of the gender score

Variable name	Coefficient estimates	***p***
TICS	0.63	< 0.001
Family status 2009–2014	0.52	< 0.001
Risk-taking behaviour	0.37	< 0.001
BFI: Agreeableness	0.31	< 0.001
BFI: Neuroticism	0.27	< 0.001
BFI: Extraversion	0.27	< 0.001
UCLA-Loneliness	0.44	< 0.05
BFI: Conscientiousness	0.17	< 0.05
Education	-0.04	< 0.05

Logistic regression models using the identified variables from PCA were calculated, and non-significant variables were removed one by one in a descending order. Finally, the above model was reached with nine independently significant variables. The area under the curve statistic was .795, *p* < .001, 95% CI [.769, .821] which indicates fair-to-good sensitivity/specificity (test values ranged between 0.5 and 1) (*n* = 1869)

### Calculation of gender score in individual cases and distribution between females and males

The coefficients of the final logistic regression analysis were used to calculate the gender score with a range from 0, extremely masculine, to 100, extremely feminine (Fig. 2). The gender score distribution in females and males differed: 55% of males were in the first tertile with more masculine gender scores and 51% of females were in the third tertile with the more feminine scores. At the same time, the distributions showed considerable overlap with a 36% and 31% of females and males in the middle tertiles and 13% of females and 14% of males having score values of the opposite expected gender (Fig. 2, Table S2).

The area under the curve statistic for 1089 participants who had a gender score available was .795, *p* < .001, 95% CI [.769, .821] (Fig S1) which indicates fair-to-good sensitivity/specificity to separate women from men.

### Association of gender score with clinical and psychosocial variables

In the third step, we performed linear regressions to further investigate associations between sex, the gender score, and biological and well-being variables that were not used to build the score. A linear regression model was performed to determine the impact of sex and gender in combination (Table 4). These models revealed that sex was significantly associated with LDL-cholesterol and total cholesterol, with the gender score not showing any additional association in the models. In contrast, the gender score was significantly associated with cortisol levels, CES-depression, negative affect, and life satisfaction, with sex not associated with these parameters. Interestingly, hand grip strength was associated with both sex and gender score. The associations with hand grip strength, cortisol, and life satisfaction were negative, suggesting that a more feminine score is associated with lower levels of these parameters, whereas all other parameters were positively associated.

**Table 4:** Prediction of clinical and psychological variables by sex and gender score in combined linear regression models

Dependent variable	Female sex	Gender score	Female sex	Gender score	*R*^2^
	β	*p* value	β	*p* value	
LDL-Cholesterol	13.69	**< 0.001**	− 0.043	0.381	0.033
Total cholesterol	25.023	**< 0.001**	− 0.059	0.273	0.088
HbA1c	− 0.069	0.107	− 0.001	0.287	0.007
CES-Depression	0.246	0.576	0.057	**< 0.001**	0.068
Cortisol	− 15.016	0.067	− 0.530	**0.001**	0.027
Hand grip strength	− 15.601	**< 0.001**	− 0.019	**0.021**	0.654
Negative affect	− 0.462	0.485	0.058	**< 0.001**	0.023
Life satisfaction	1.200	0.078	− 0.120	**< 0.001**	0.081

We used linear regression models to determine the association of sex and the gender score with biological and psychosocial variables. For each of the biological and psychosocial (dependent) variables, a linear model was calculated, all including sex and the gender score as independent variables. All models explained less than 10% (*R*^2^) of the variability, with the exception of the model for hand grip strength, in which 65% of the variability in grip strength was explained by sex and the gender score. β*, unstandardized regression coefficient beta (*n* = 1045)

## Discussion

Our study constructed a gender score in a retrospective manner from available study variables that characterized women and men based on only sociocultural variables, covering the four dimensions of gender. Biological sex and gender score characterized participants differently and had different predictive power for a number of clinical and psychological variables. The results will allow us to explain a greater part of variability among women and men during aging, and they provide researchers with clinical databases with a template to include gender in a retrospective manner in their analysis.

We used the BASE-II cohort that provided data on older, predominantly healthy adults in and around Berlin, Germany [21], to construct a gender score in analogy to the score that was developed in a mainly Canadian cohort of younger patients with acute coronary syndrome and by others in the Canadian health database [17, 18]. The novelty of our approach was the fact that we developed the gender score in a completely retrospective manner in a non-working cohort that undergoes systematic follow-up investigation. BASE-II was not primarily designed to analyse the impact of gender on study data [12]. This retrospective design was limited by the fact that not all the desired gender-related variables were available and not all dimensions of gender could be covered with similar strength. However, it has the advantage that the variables are measured prior to the outcome, meaning that the health status at the time of follow-up does not influence the components of the gender score.

As expected, more than half of females and males clustered in the respective feminine and masculine tertiles. However, the distribution showed a considerable overlap with almost a third of females and males in the middle tertiles and 13% of females and 14% of males having score values of the opposite gender. Thus, the gender score is able to separate women from men with a good sensitivity but clearly differs from biological sex, contributing additional explanatory power and is a major advantage of this score. The distribution is similar but somewhat different from the distribution found by Pilote in their younger cohort with myocardial infarction. They found a more asymmetrical distribution with a stronger clustering of males in the masculine area and a broader distribution of females over the whole scale, indicating that males kept their masculine characteristics whereas females had acquired more masculine characteristics. In contrast, more of our males were found in the middle tertile. This may be an effect of age or retirement—it is likely that the work-related criteria in our older cohort were less important.

When constructing the gender score in our study, all four dimensions of gender were covered. This was a much broader coverage of gender dimensions than in the only directly comparable retrospective studies [17, 18]. They are based on larger cohorts from Canada, therefore not directly comparable with Europe, include a younger working population and are heavily based on work-related variables, therefore not suitable for our retired/aged population, and are both purely cross-sectional, whereas the gender score in our longitudinal cohort study will allow us to develop risk prediction models. The variables family status and education, reflecting gender relations and institutionalized gender, had a high impact in gender score calculation, underscoring the significance of this area.

Thus, in the aged cohort of BASE-II, with mainly retired persons, other variables had a strong contribution to gender than in younger working cohorts. Psychological parameters, such as perceived stress, loneliness, agreeableness, and neuroticism, contributed significantly to the psychosocial differences between women and men. We conclude that the construction of a retrospective gender score should be based on the data of the study itself. In addition, it may be difficult to transfer a retrospective gender score from one study to another. This means that a gender score should not be taken from the literature and used as a fixed algorithm for a study population, but rather be constructed based on as many suitable study parameters as possible and then applied only to the probands of that study. Comparison of the main variables in gender scores from different studies will allow the assessment of the dependency of gender-related parameters and gender constructs from external variables such as age, ethnicity, cultural background, and working conditions and hopefully identify the most robust ones.

### Use of gender score in prevention

The association of the gender score with clinical and psychosocial variables and risk factors was already tested by Pilote and Lacasse. Lacasse claimed that associations between gender index scores and presumed gender-related variables identified a priori and not included in the gender index supported the validity of the construct [17]. We followed these approaches and tested the association of the gender score with clinical and psychosocial variables. In combined linear regression models, gender score, but not sex, was significantly associated with cortisol levels, CES-depression, negative affect, and life satisfaction. Following the arguments of Lacasse, this would support the validity of our construct. Nevertheless, this must be confirmed by external validation in future studies.

Pelletier et al. found in their prospective approach that a higher gender score, but not female sex, was associated with an increased risk of hypertension, diabetes, family history of cardiovascular diseases, and increased depressive and anxiety symptoms [3]. They concluded that traditional sex differences in risk factors may partly be explained by patients’ personality traits, social roles, and life context. If this is true, tackling these risk factors and traits in gender sensitive prevention projects could reduce the risk for cardiovascular diseases. Previous investigations have shown that prevention programs need to be designed in gender sensitive manner to reach their respective audiences. Therefore, when focusing on target groups in prevention programs, not only the biological characteristics should be used but also the elements of the gender score as identified in our project [34].

### Limitations of the study

In this paper, we aimed to generate a gender score that allows us to assess whether gender, as an assembly of sociocultural parameters taken together, affects disease risk differently in women and men. Undoubtedly, there are also limitations associated with this approach. Using individual variables related to gender, such as living alone or anxiety, would enable a study to identify specific factors of importance which, independent of sex, affects health outcomes. A compositional variable such a gender score cannot in itself identify such key factors. However, as compared with the use of a set of gender-related variables, a single score provides greater statistical power by reducing the number of covariates included in multiple regression models, offer the possibility to test interaction terms, and reduce multiple comparisons [15, 16, 35]. Therefore, the gender score makes it possible to capture better the interaction of gender with other variables.

When constructing a gender score, we needed to consider that gender, as a social construct, shifts over time and between generations, differs between places and cultures, and depends on socioeconomic conditions. Ideally, the selection of variables for a gender score would be contextual, both in terms of time and place. Therefore, our approach to develop a gender score specifically for the cohort in which it should be used has its strength. At the same time, this leads to significant limitations: the limited availability of variables may explain differences in the variables that constitute the gender score between our study and previously published prospective data [3]. We must accept the available variables and cannot, in a prospective manner, select the best ones. This is, however, an inherent component of the retrospective approach which is justified by the aim to develop a method that enables researchers to measure gender in a retrospective manner. Such an approach is valuable since many studies exist in which an estimate of gender, even if retrospective, could uncover new aspects and reveal important insights.

## Conclusions and future aspects

With our strategy, we were able to develop a gender score in a retrospective manner in an elderly, non-working European cohort. This means that the notion of gender can be retrospectively introduced into a large number of studies and contribute to explain differences between women and men in health outcomes. In calculating gender scores in a number of different cohorts from different cultural background, ages, and ethnicities, we will learn which variables are most robust predictors of gender throughout cultures. Furthermore, if in future studies, when longitudinal data will become available, gender turns out to be correlated with risk factors or outcomes, this will provide new aspects for focused prevention to the health care systems.

## Supplementary Information

**Additional file 1: Figure S1**. ROC analysis with 1089 participants for gender score and sex variable. **Table S1**. Correlation between gender variables in PCA. **Table S2**. Gender scores in the different tertiles of our cohort.

## Data Availability

Due to concerns for participant privacy, data are available only upon request. External scientists may apply to the Steering Committee of BASE-II for data access. Please refer to the BASE-II website (https://www.base2.mpg.de/7549/data-documentation) for additional information.

## Abbreviations

BASE: Berlin Aging Study
BASE-II: Berlin Aging Study II
BMBF: Bundesministerium für Bildung und Forschung
CES-depression: Center for Epidemiologic Studies Depression Scale (CES-D)
CIHR: Canadian Institutes of Health Research
GENESIS-PRAXY: Gender and sex determinants of cardiovascular disease: from bench to beyond-premature acute coronary syndrome
HbA1c: Haemoglobin A1C
LDL: Low-density lipoproteins
