# Supplementary material for: Gender score development in the Berlin Aging Study II: a retrospective approach
Source: Biol Sex Differ. 2021 Jan 18;12:15. doi: 10.1186/s13293-020-00351-2 (PMC7814714; doi:10.1186/s13293-020-00351-2)

Supplement

Methods

Inclusion of gender variables from BASE-II

Family status contrasted “What is your marital status?” with possible answer options as (1. Married 558

lives with partner, 2. Married lives separately, 3. Single, 4. Divorced, 5. Widow). Risk taking was 559

assessed as: Are you in general a person who is willing to take risks or do you try to avoid them? 560

(PRISK) (How much are you willing to take risks: 1– when driving (PRISK2), 2– when investing money 561

(PRISK3), 3– when doing sports or having free time (PRISK4), 4– in your professional career (PRISK5),

5– with your health (PRISK6), 6– with your trust in strangers (PRISK7), 7– after winning lottery? 563

(PRISK8). Perceived stress was assessed with 10 items from the Perceived Stress Scale (Cohen, 564

Kamarck, & Mermelstein, 1983), answered on a scale ranging from 0 (never) to 4 (very often). 565

Chronic strain was assessed with eight items selected from the Trier Inventory for Chronic Stress 566

(Schulz, Schotz, & Becker, 2004) answered on a scale ranging from 0 (never) to 4 (very often). The big

five personality dimensions were assessed using three items each, selected from the German short 568

version of the Big Five Inventory (for details, see Mueller et al., 2016). Items were answered on a 7-569

point Likert scale ranging from 1 (strongly disagree) to 7 (strongly agree). Loneliness was measured 570

using seven items from the UCLA Loneliness Scale (Russell, Cutrona, Rose, & Yurko, 1984), rated in a

scale from 1 (does not apply at all) to 5 (applies very well). Employment status was assessed using 572

Are you currently employed? (Yes/No). Education was measured as number of years spent in formal

schooling. The item the good things in my life are determined by other people (Gerstrof D et al. 574

2019, Kunzmann et al. 2002, Levenson et al. 1981) was rated in a scale from 1 (does not apply at all) 575

to 5 (applies very well). Life satisfaction was assessed with three items selected from the 576

Philadelphia Geriatric Center Morale Scale (Lawton, 1975) rated on a scale from 1 (strongly disagree)

to 5 (strongly agree). Responses to the items (e.g., “I sometimes feel that life isn’t worth living”) were

reverse coded so that higher scores indicated higher life satisfaction. Negative affect was assessed 579

with three items about the frequency of experiencing particular emotions in the last year taken from

the Positive and Negative Affect Schedule-X (Watson & Clark, 1994). Items were rated on a scale 581

from 1 (not at all) to 5 (very often). Items included feeling sad, drowsy, and sluggish. 582


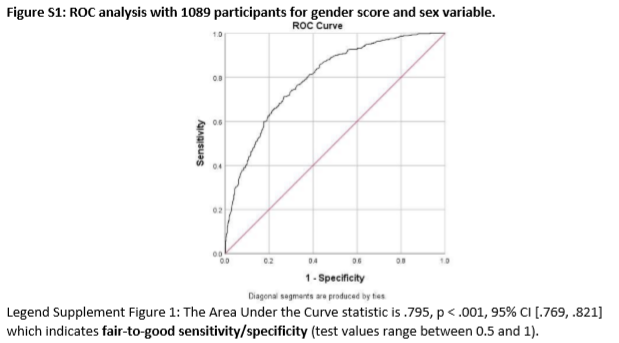


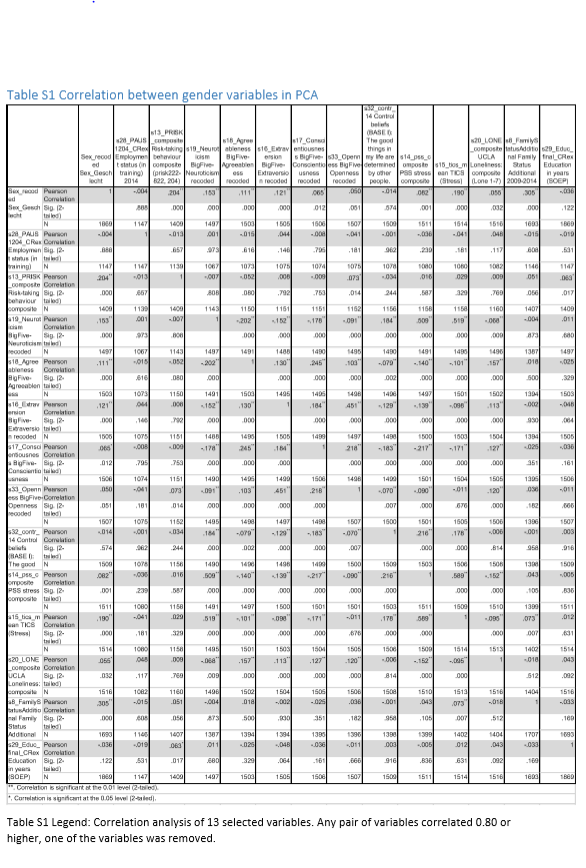


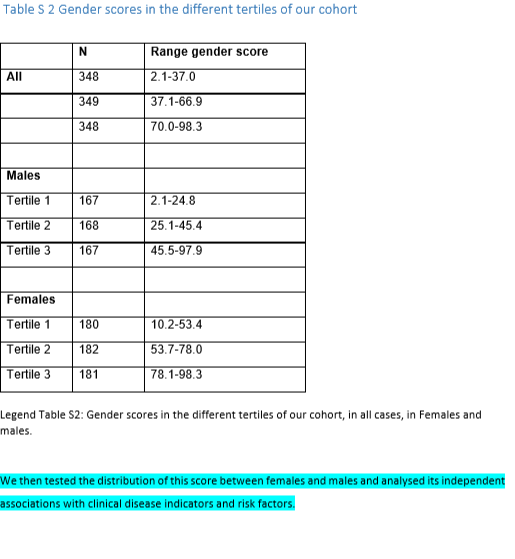

Supplement: Supplementary file 1 — Additional file 1: Figure S1. ROC analysis with 1089 participants for gender score and sex variable. Table S1. Correlation between gender variables in PCA. Table S2. Gender scores in the different tertiles of our cohort. [file 13293_2020_351_MOESM1_ESM.docx]
